# Influences on participation in a programme addressing loneliness among people with depression and anxiety: findings from the Community Navigator Study

**DOI:** 10.1186/s12888-020-02961-x

**Published:** 2020-11-26

**Authors:** Johanna Frerichs, Jo Billings, Nick Barber, Anjie Chhapia, Beverley Chipp, Prisha Shah, Anna Shorten, Theodora Stefanidou, Sonia Johnson, Brynmor Lloyd Evans, Vanessa Pinfold

**Affiliations:** 1 McPin Foundation, 7-14 Great Dover Street, London, SE1 4YR UK; 2 Division of Psychiatry, UCL, 149 Tottenham Court Road, London, W1T 7NF UK; 3 Complex Depression, Anxiety & Trauma Team, St Pancras Hospital, London, NW1 0PE UK

**Keywords:** Loneliness, Co-production, Qualitative research, Anxiety, Depression, Social inclusion

## Abstract

**Background:**

Loneliness is associated with negative outcomes, including increased mortality and is common among people with mental health problems. This qualitative study, which was carried out as part of a feasibility trial, aimed to understand what enables and hinders people with severe depression and/or anxiety under the care of secondary mental health services in the United Kingdom to participate in the Community Navigator programme, and make progress with feelings of depression, anxiety and loneliness. The programme consisted of up to ten meetings with a Community Navigator and three optional group sessions.

**Methods:**

Semi-structured interviews were carried out with participants (*n* = 19) shortly after programme completion. A co-produced two-stage qualitative approach, involving narrative and reflexive thematic analysis, was undertaken by members of the study’s working group, which included experts by experience, clinicians and researchers.

**Results:**

The narrative analysis showed that individuals have varied goals, hold mixed feelings about meeting other people and define progress differently. From the thematic analysis, six themes were identified that explained facilitators and challenges to participating in the programme: desire to connect with others; individual social confidence; finding something meaningful to do; the accessibility of resources locally; the timing of the programme; and the participant’s relationship with the Community Navigator.

**Conclusions:**

We found that people with severe depression and/or anxiety supported by secondary mental health services may want to address feelings of loneliness but find it emotionally effortful to do so and a major personal challenge. This emotional effort, which manifests in individuals differently, can make it hard for participants to engage with a loneliness programme, though it was through facing personal challenges that a significant sense of achievement was felt. Factors at the individual, interpersonal and structural level, that enable or hinder an individual’s participation should be identified early, so that people are able to make the best use out of the Community Navigator or other similar programmes.

## Background

The topic of loneliness and how to address it has recently attracted increased attention, both in the United Kingdom and internationally. This has included high-profile campaigns such as the Jo Cox Foundation “Great Get Together” in the United Kingdom (UK) [1] and Australia’s Coalition to End Loneliness [2], and media coverage including the BBC’s Loneliness Experiment [3]. This has come about, in part, due to growing awareness of the consequences of loneliness, including how it increases mortality risk by 26%, making loneliness comparable to other known health risks such as obesity and physical inactivity [4].

Loneliness has been variously defined [5, 6] but is generally understood to be the distressing subjective state experienced when there is a gap between actual and desired social relations [7] and is not solely about being alone [8]. It is this focus on subjectivity, and the appraisal of one’s perceived relationship quality, that distinguishes loneliness from related concepts such as social isolation, which refers to a lack of contact with other people, often objectively measured by counting social ties [9].

Loneliness is often associated with older age in Western societies [10] but it is increasingly recognised that loneliness can affect people across the life course and certain groups may be particularly susceptible [11], including the unemployed, younger people and people with mental health problems [12, 13]. Indeed, cross-sectional studies have found associations between loneliness and anxiety and depression [10, 14–16], with Meltzer et al. finding an 11-fold increase in the likelihood of feeling lonely among those who are depressed [17]. Furthermore, a systematic review found that higher levels of loneliness predicts greater depression severity and lower rates of remission [18] and longitudinal work has shown that loneliness and depression are predictors of early death, particularly among older men [19]. Taken together, this highlights the need for intervention.

To date, most trials of interventions to alleviate loneliness have been carried out with older people, with the most promising approaches being those that seek to restructure maladaptive social cognitions [20, 21]. Understanding of the mechanisms that contribute to intervention success is, however, limited, and there have been calls for more research, particularly qualitative research, to investigate how such interventions are affected by experiential and contextual factors [22, 23].

Within mental health, four types of loneliness intervention strategies have been proposed: changing cognitions; social skills training and psychoeducation; supported socialisation or having a ‘socially-focused supporter’; and ‘wider community approaches’ including social prescribing [9]. A systematic review found preliminary evidence that interventions involving cognitive modification may also be effective for people with mental health problems, but suggested further evidence was needed [24]. As approaches to address loneliness among people with mental health problems are further developed and assessed [25], it is important that their evaluation involves consideration of not only whether, but also how and why, the intervention may work.

This paper presents a co-produced qualitative analysis of the factors that appear to influence participation in the Community Navigator programme, which was developed to improve community connections and reduce feelings of loneliness among people with severe depression and/or anxiety [26]. By looking at service users’ experiences of the programme, we aimed to understand in more detail what affects people’s ability to embrace loneliness interventions and explore potential reasons why programme effects differ between individuals as well as the experience and acceptability of that intervention to different population groups.

## Methods

### The Community Navigator Study

This paper presents data from the Community Navigator Study, which was conducted over two years in two London NHS Trusts and was funded by the NIHR School for Social Care Research. The study involved three phases: development of the programme using a co-production approach [27]; preliminary testing of the programme with ten service users with severe depression and/or anxiety on the caseload of one of two community mental health teams; and a feasibility trial of the programme involving 40 service users from the same community teams. A full description of the Community Navigator programme [26], and results of the feasibility trial have been published [28].

Service users taking part in the programme were typically severely anxious and depressed. At baseline, the mean score for all participants on the GAD-7 measure of anxiety and the PHQ-9 measure of depression, exceeded clinical thresholds for severe symptoms [29, 30].

To help address this, service users were offered up to ten sessions with a Community Navigator, as well as three group sessions. In the individual sessions, Community Navigators used a structured network mapping process to help participants identify people, places and activities which were important to them, and then helped participants to set goals and enact plans to improve the quantity or quality of their social connections [31]. The Community Navigators used a solution-focused approach. The programme also included three group “meet-up” sessions, where participants could meet, share information about local resources and social groups, and discuss strategies to increase social connections. A budget of up to £100 per participant was available to facilitate developing new social connections including accessing groups and activities. Community Navigators were not required to have mental health qualifications (although one did). Instead recruitment focused on their local knowledge, work or informal experience in roles which involved encouraging community participation, and good interpersonal skills including empathy, communication and a positive, forward-looking attitude. The Community Navigators were employed by an NHS trust and received monthly group supervision from qualified mental health professionals (social workers and an occupational therapist) in the community mental health teams in which they were employed.

### Co-producing the study

Co-production involves people with different expertise, including mental health service users, working collaboratively throughout all stages of a research project [27] as partners. This study brought together experiential, practitioner and research expertise, through a working group of 12 people, including: experts by experience who had personal, lived experience of depression, anxiety and loneliness; mental health practitioners; and researchers.

People in the working group could have more than one type of ‘expertise’ and identify with multiple roles. The working group co-designed the content of the programme, recruited and helped train the Community Navigators and co-developed the research procedures. The working group members also collaborated to co-produce the analysis for this paper, following co-production principles including shared decision making [32].

### Sample

Initial contact was attempted with all service users who had met a Community Navigator for at least one session during the feasibility trial, apart from with one participant who had died. A researcher contacted service users by telephone, shortly after their final programme session, inviting them to take part in an interview about their experiences of the different aspects of the programme and its perceived impacts. The researcher explained the interview process, including that they also had experience of depression and anxiety, following a peer research methods approach [33]. This approach can be helpful in addressing power imbalances, acknowledging the importance of identity and role status in psychiatry, as well as following up on areas that researchers without personal experience of mental health issues might not. Where service users were interested in taking part, they were sent an information sheet and a meeting with the researcher was arranged.

### Data collection

Semi-structured interviews took place between November 2017 and March 2018 and lasted around 40 min (range 21–73 min). The interview guide was piloted in the preliminary testing phase with ten people, and modified as a result, with input from the working group. Interviews were arranged either in the service user’s home (*n* = 14), or at the community mental health team (*n* = 4). One interview was conducted over the telephone. Most interviews were audio-recorded (*n* = 17), and in the two cases where permission to audio record was not given, detailed notes were made and checked with the person at the end of the interview. All interviews involved a one-to-one interaction between the service user and researcher, apart from in two cases: one where the housing support worker was present and one where the service user’s child (under 5) was present. Participants were offered £20 as a token of thanks for their time. Following each interview, the researcher made field notes, reflecting on their experience and noting contextual information that would not be captured through audio recording, such as how the interviewee appeared and information about the interview setting.

### Data analysis

A multi-method qualitative approach to the analysis was undertaken, using both a narrative analysis of three case study interviews [34, 35], as well as reflexive thematic analysis [36, 37] of all 19 interviews. The narrative analysis allowed us to explore specific cases in-depth, complemented by the breadth brought by the thematic analysis of the whole data set. Adopting this approach enabled us to look in detail at individual journeys through the programme, while also considering the influences on participation across the service user group interviewed.

The narrative analysis involved three stages:

1. Three interviews were purposively selected for maximum variation in terms of the progress service users were able to make within the programme. Progress was determined qualitatively, in terms of the extent to which participants reported going out and engaging in social activities, and feeling less depressed, anxious and lonely, during their interview. The sampling was led by the researcher who conducted the interviews, supported by wider team discussion.
2. Six members of the working group, including four experts by experience and two researchers met three times, once for each of the sampled interviews. Prior to each meeting, the six members each read all the interview transcripts and made detailed notes on their understanding of the different influences on the individual’s journey through the programme and how their narrative was told. At each of the three meetings, working group members described their own interpretation of the narrative. These were discussed by the group and notes were taken to create a collective summary of each ‘case’. The summaries were then circulated to the group for further input and agreement [38].
3. Drawing on these summaries and the field notes which were taken immediately following the interviews, the case studies were then written up for inclusion in this paper. When writing the case studies, consideration was given to *how* the participants spoke about their experience of the programme, including non-verbal information (from the field notes) and the way the narrative was constructed, as well as *what* the participant said about the programme. This approach has been argued to offer a rich description of how a participant experiences a situation over time [39].

**Fig. 1:**
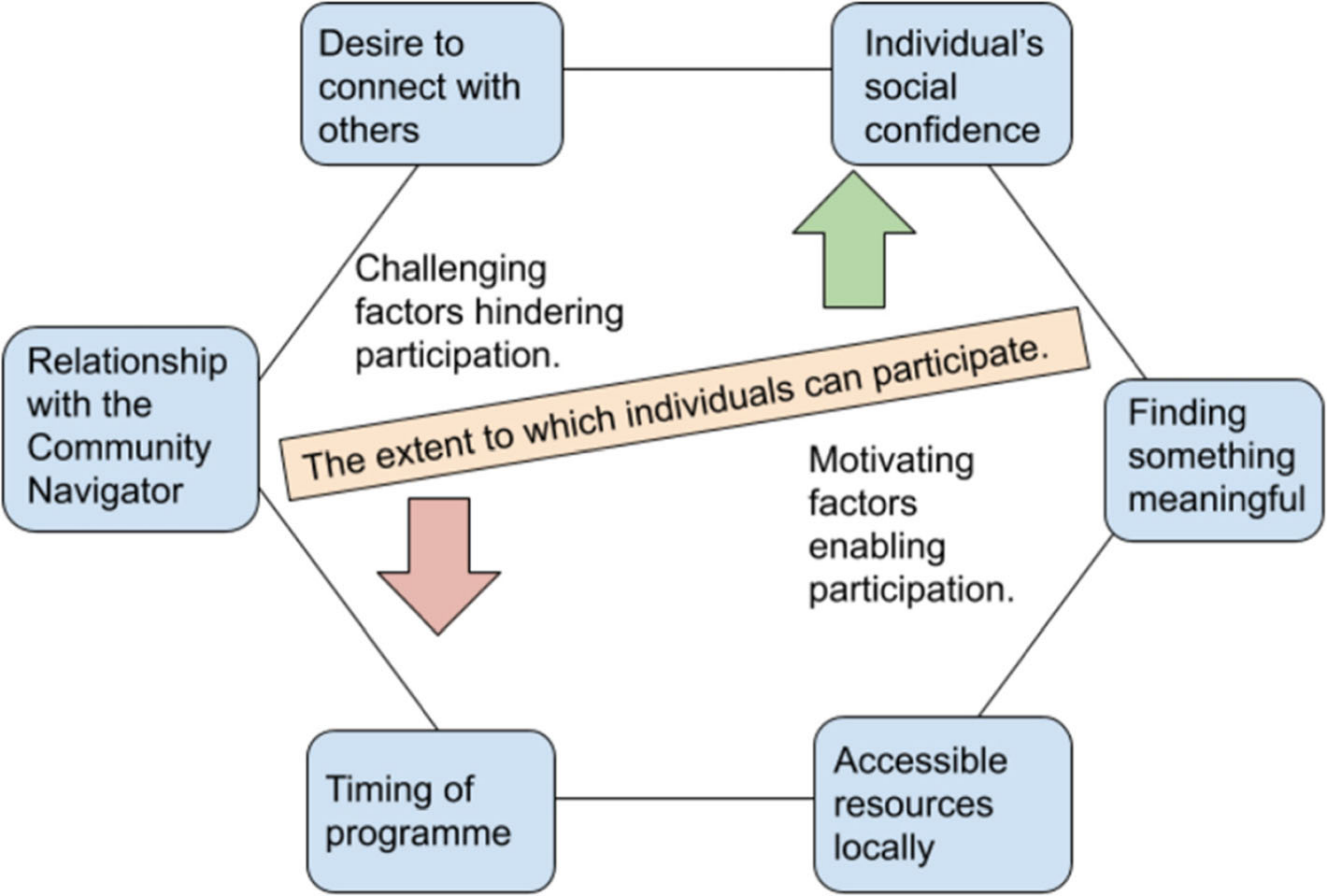
Influences on participation in the Community Navigator programme

A reflexive thematic analysis was conducted, informed by the approach outlined by Braun and Clarke [40]. This involved:

1. Familiarisation: All interviews (*n* = 19) were read by one or more members of the working group (*n* = 10), of whom five were employed as researchers, four involved as experts by experience and one was a practitioner. Some people brought expertise from more than one category.
2. Initial coding generated: Working group members collaboratively generated a list of main codes and sub-codes from their reading of the transcripts, focusing on the influences affecting people’s participation in the programme. This process was inductive and considered both the explicit (semantic) content of the interviews as well as the underlying (latent) ideas and meaning. A researcher coded used these codes, as well as additional codes identified while carrying out the coding, on all 19 interview transcripts, facilitated by NVivo 11 software.
3. Searching for themes: Codes were examined to generate higher-level themes. These were then reviewed in an iterative process that involved going between the data extracts assigned to each code and initial themes, until final themes were reached.
4. Reviewing themes: These themes were discussed, refined within the working group.
5. Defining themes: The final wording of themes were agreed by the working group, and definitions provided to ensure accurate application of each.
6. Write up: The themes were written up, supported by illustrative quotations from participants and a diagram that was co-designed within the group (see Fig. 1).

## Results

The findings are presented in two sections. All names used are pseudonyms and some key details, such as place names or activity descriptions, have been changed or omitted to ensure anonymity.

### Participant characteristics

Of the 29 participants who met their Community Navigator at least once, 19 people provided written consent to take part in interviews about their experiences of the programme; their demographic characteristics are shown in Table 1. Ten other people who participated in the programme did not take part in the qualitative interviews. The reasons for this were: declined (*n* = 7), unable to make contact (*n* = 2) and death (*n* = 1).

**Table 1:**
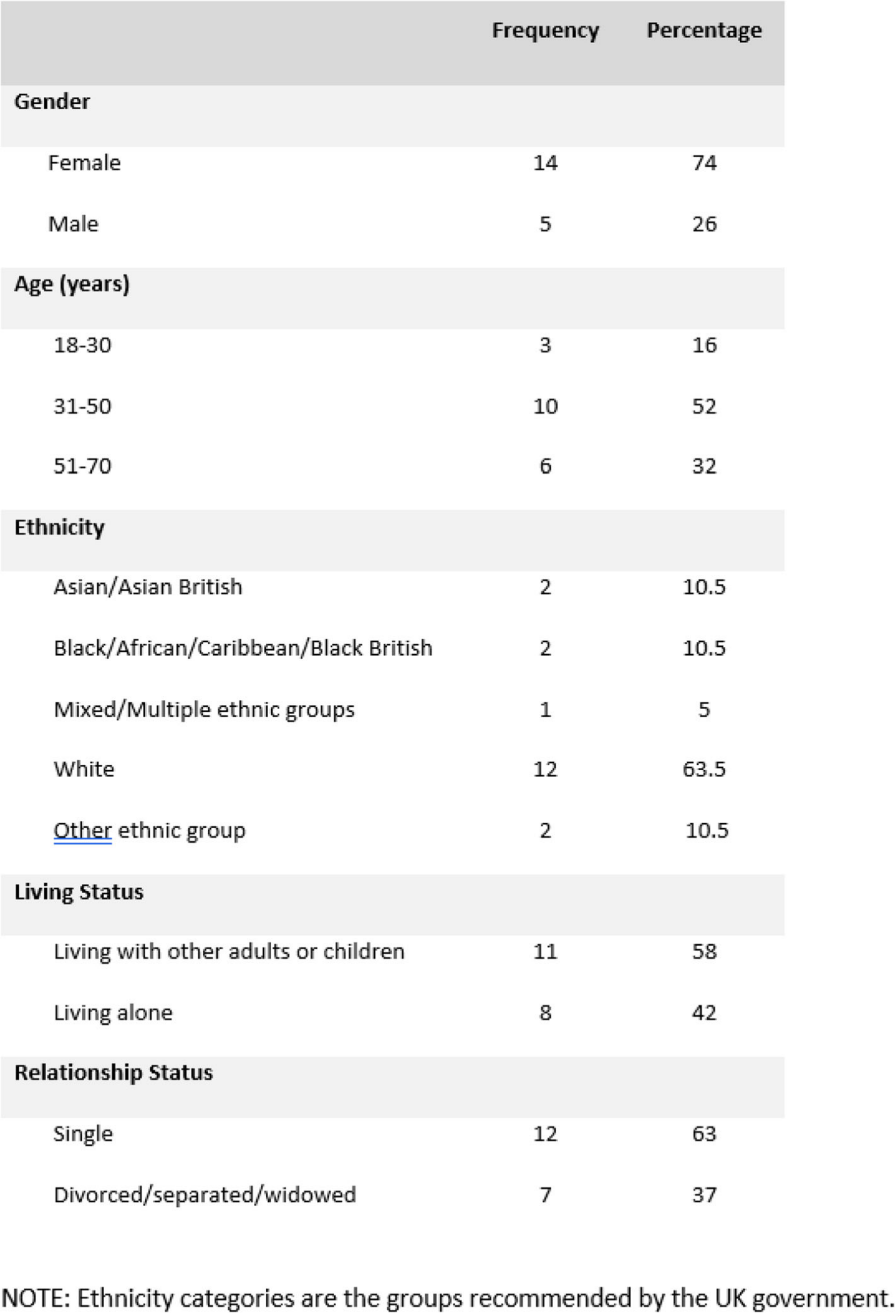
Demographic characteristics of interviewees (*n* = 19)

### Narrative analysis

Narrative analysis was used to explore how three individual service users experienced and made sense of their journeys within the programme. The interviews were selected to provide contrast in terms of the progress made: one person had a renewed sense of hope and developed new social connections, one person felt more confident leaving home and had tried out some groups but reported no changes to their social network and one person attended meetings with a Community Navigator, but found it hard to make progress.

#### Case study 1: Regaining hope, self-confidence and sociability

Leah, a single mum, had previously been admitted to hospital for treatment for depression: “*I completely zoned out, became a zombie”*. When she was discharged, she told us that she became socially anxious, wearing hooded tops to hide her face and making no eye contact with anyone when she went out. She *“felt ready”* for things to change but was not sure how to go about doing so.

As Leah moved on to describe her experiences of the Community Navigator programme, the tone of her narrative changed to one of optimism and energy, and it is clear that her relationship with the Community Navigator was at the heart of this. In an animated voice, with upbeat music playing in her home, she explained how she got on with her Community Navigator from the start, forming a close relationship that was *“almost like a friendship”.* She contrasted this to her relationship with family members who could be critical and dismissive: *“I’m always reminded [by them] of the mistakes I’ve made”.* She described how her Community Navigator had always been positive, encouraging her to do the things she wanted to do and affirmed her strengths: *“[they were] the one to praise me.”* Leah’s repeated juxtaposition of these relationships indicated how important her connection with the Community Navigator was in enabling her to get the most out of the programme.

Together the Community Navigator and Leah tried out a number of activities that fitted with her interests. Leah was open to these suggestions: “*let’s go for it*”, pushing through any initial nerves about attending and spoke with enthusiasm about the activities being “*great fun*”. As she was naturally *“talkative”*, she told us that she made friends along the way, some of whom she continues to be in touch with *“all the time”*. This natural sociability came across in the interview: the researcher’s field notes recorded that Leah was welcoming, engaging and chatty, being interested in the researcher’s life, asking questions and offering advice.

Taking part in the programme gave Leah a sense of *“hope”* and enabled her to *“start believing in [herself] again”,* things that she had lost during her depression. Though Leah talked about continuing to feel lonely occasionally, especially when *“in bed at night alone”*, she optimistically described her plans for the future, portraying a strong sense that she will continue “*making [herself] happy”*, building on everything she got out of the programme.

#### Case study 2: Progress addressing fears about going out but no new social connections forged

At the heart of Anita’s narrative was exploration: “*I wanted to get to know and go and see places”*. She had recently escaped a traumatic situation and before joining the programme, spent much of her time alone at home, cut off from the world. For Anita, the Community Navigator programme had been an opportunity to explore her interests and the local area, in a way that felt safe and manageable: “*I just listened to what [the Community Navigator] said, and [they were] so calming, and I went and did it”.*

Despite progress in going out, Anita still found she was “*a bit nervous about meeting new people*”. Much of what she did as part of her programme was only done with her Community Navigator. The one time she did attend a social group, she was *“surprised”* at herself for striking up conversation with someone else in attendance. Yet her continuing struggles with social contact were apparent: the researcher recorded that while Anita was friendly and engaging, she also seemed nervous. This was reinforced by what was said during the interview *“My heart is going like this, just talking to you”.*

At the end of the programme, Anita reported that she had no new social contacts and had not improved relationships with existing contacts. Nevertheless, she said that she no longer felt lonely because she knew more about what to do in her local area and had the confidence to go out: *“There’s so much to do, I can go out my front door…no, I don’t feel lonely”.* Anita also reiterated that *“I did it”*, indicating her sense of pride and achievement at having challenged herself by going out. In terms of what Anita would do from here, at times in her narrative she talked with full commitment *“I’m going to carry on”,* but at others she was more hesitant: “*I would love to, it’s just making that first initial move…I don’t know how to get past that yet”.* We were left uncertain about whether Anita would continue going to the places she had discovered with her Community Navigator.

#### Case study 3: Struggling to engage with the Community Navigator programme

Arjun’s whole narrative was imbued with a sense of despondency. Though he entered the programme hoping he could find places to go beyond his home, as he did not “*get out much*”, he found it difficult to participate. His low mood, coupled with sleeping problems, made going out and even opening the door to meet with the Community Navigator feel *“exhausting”.* Arjun’s continuing struggle with depression was evident throughout the interview. Much of the time his tone was flat and it was noted that he smiled rarely, leaving the researcher with a strong sense of Arjun’s feelings of emptiness.

Arjun found it particularly difficult to muster the energy to do something when it was further away or *“it just didn’t interest”* him. His repeated use of the phrase *“[they were] trying to get me to go to different places”* was very telling, suggesting that he looked to his Community Navigator to put forward ideas about what to do, as he could not identify anything he connected to. The one thing that Arjun was very clear about, however, was that he did not want to meet other people, as this made him *“feel really anxious”*. The researcher got a sense of Arjun’s anxiety at meeting someone new, recording in field notes that Arjun seemed nervous, gave little eye contact and kept his answers short. We therefore wondered whether a programme targeting loneliness was right for Arjun at this time.

Overall, Arjun’s said that he did not “*think anything [was] different*” as a result of taking part. Yet, when we look at his whole account, we see that he had expanded his boundaries by travelling *“further away”* to a place that he had never been to before, in line with his aim at the beginning of the programme. Therefore, though his story may not be one of transformation, we can see there were small steps of progress, even if he did not recognise them.

### Thematic analysis

This section builds upon the insights gained from the individual case studies to explore what influenced participation in the Community Navigator programme across the wider group. Six common factors were identified, summarized in Fig. 1. Each of these factors could challenge or motivate individuals to participate, with the relative influence of each factor differing between individuals and over time. In addition to these factors, everyone taking part in the programme was managing mental health problems, namely severe depression and/or anxiety. The influence of mental ill-health is most explicitly captured within the ‘timing of the programme’ factor, but as mental health problems were an ever-present influence impacting all other factors, they were not included as a separate theme.

#### Desire to connect with others

The interviews revealed that people had conflicting feelings about connecting with others. On the one hand, there was recognition of the benefits of socialising, and it was something that some people longed for, but at the same time, there was the feeling that relationships can be difficult to manage and open you up to feeling let down. This sometimes made it feel preferable to be alone or to keep a limited social network of those you trust.

*“…loneliness is not really good for your health, is it, your mental health or whatever. But the thing about me is maybe in a way I isolate myself, if that makes sense, because a lot of the time I feel like I can't really be around people.” (*CN04)

*“I don’t want anybody to be around me, but at the same time I do want people who care about me too, if it makes sense? I don’t know how to describe both feelings in one go. Don’t come too near to me, but care about me as well.”* (CN21)

This tension operated more or less for everyone in the programme. The conflicts were stronger, for instance, in those for whom being hurt in relationships was particularly salient and those participants tended to express greater ambivalence about connecting. In some cases, this created resistance, making it difficult for Community Navigators to work with service users to make changes to their social network.

*“Do I want to change? No…Absolutely not…No. I find people testing, boring and they always disappoint me, so, no, that’s just stress”* (CN11)

Yet everyone had agreed to join a programme about social connections and loneliness - indicating that at some level there was desire for change. Having built a trusting relationship with the Community Navigator, or having met someone at a group to whom a person related, people’s desire to connect could grow.

*“[Name of Community Navigator] helped me in the fact that [they] made me try to see some people differently to what I may initially... not to just initially cut everybody off from the start without giving it a chance and seeing whether we would get on.” (*CN03)

#### Individual’s social confidence

How confident a person was in their own social skills was an important influence on the programme. The Community Navigators worked with people who described themselves as “*a bit of a chatterbox*” (CN39), and had not previously had difficulty connecting with other people, but equally people who believed they were “*not very good*” (CN03) socially and found the thought of being around other people uncomfortable and anxiety provoking.

*“I don't socialise a lot. I was very scared of meeting people. I have anxiety of meeting new people that I don't know.”* (CN32)

*“I still find that difficult, talking to people I don’t know. My heart is going like this, just talking to you and I’m having difficulty trying to explain to you.”* (CN29)

Individuals’ confidence varied depending on the social situation. Group settings were seen as particularly daunting, especially those that required active rather than passive engagement, or were in places that were busy and unfamiliar. It also depended on how comfortable the person felt around the group facilitator and other group members, with people who did not think of themselves as socially skilled surprising themselves in the right environment.

*“The place was really busy, you know, there was lots of people outside chatting and inside chatting and laughing and whatever, and it was, like, I didn’t really want to have to walk straight in to that group of people, it made me really nervous. So yes, I kind of chickened out on that one.”* (CN25)

*“I guess in groups I feel uncomfortable around I talk less, but I felt fine around these people, they seemed to be very nice and approachable and I ended up talking a lot more because of it.”* (CN25)

Where individuals were more confident, they often found it easier to go out and try different social groups. For those who were less confident, it sometimes took more time and required greater encouragement from their Community Navigator, as well as the person’s own determination to challenge their anxieties. Being able to be accompanied by their Community Navigator was particularly useful in helping people to feel able to enter social situations, interact with others, and gain the confidence needed to do it again by themselves in the future.

*“You know, [name of Community Navigator] came with me, and we sat there for a cup of tea, and it wasn’t so scary. It wasn’t scary. Like, you know, it’s the fear of going in there. Do you know what I mean? And pulling up, it’s a big building, and you go in there… And, you know, I’m going there on my own – something I would never have done, never.”* (CN31)

#### Finding something meaningful

The programme involved Community Navigators working with service users to identify opportunities that would bring them into closer contact with other people, such as attending activity groups or a community centre. Where service users had existing interests or had enjoyed activities in the past, it was easier to progress to trying out opportunities than where people did not feel they could connect to anything, even when presented with options.

*“Well I think I asked [name of Community Navigator] and told her that I loved animals and they told me about [name of a farm] around here, which I’d never been to, I’d never heard of, even.”* (CN29)

“*Then I looked at the list of projects that are around and I didn’t really feel like I could relate to anything and whether it’s because they didn’t have enough information or what, or if it’s just stuff I could have found out on the internet myself, I don’t know*.” (CN26)

For these loneliness-reducing opportunities to become embedded into a person’s everyday routine, it was important that they felt meaningful. Meaning was derived in different ways. For some, it came from developing new skills, or through an improved sense of wellbeing, while for others it was through doing something that helped others. The Community Navigator’s skill in helping to identify values was key. Engagement was also linked to self-confidence and feeling competent, and where people did not feel they were that skilled enough at the activity, it often hampered their enjoyment and sustained engagement with it.

*“I’m really getting into the meditation and relaxation. I think it really is good, really beneficial. I come out of there feeling really chilled.”* (CN12)

*“Well it made me have more insight because I'm not very computer literate so it's a new learning curve for me.”* (CN03)

*“It was okay but I don’t think I, myself, was very good at it. I don’t think there were many questions I could really answer myself.”* (CN04)

A particularly important influence was feeling that the other people attending such activities were in some way similar. This could come from a shared identity, for instance, having the same health problem, belonging to a similar demographic, or sharing an interest. Where people attended several activities but could not relate to the other people, it could be demoralising and reinforce a sense of loneliness.

*“Doing something like the [artistic] course, I could feel the difference already because there are people there of a like mind, people that are creative.”* (CN35)

“*Maybe I just didn’t really feel like I fitted in. Maybe it was similar to before, I didn’t feel like the people I was around were really my age or people that I’d really have a social life with.”* (CN04)

*“It was interesting to meet other people in similar situations to me. To hear about their experiences and what they’d been through. How they were dealing with things.”* (BA15)

The programme also involved the Community Navigators working with individuals to set person-centred goals. This could create meaning for people, giving them a sense of direction and feeling of achievement as goals were completed. However, for others, who found it difficult to identify goals, or goals that seemed manageable, it could add to a sense of burden and a feeling of failure.

*“Because I had the goal always in my head to do the floristry course and that pushed me. It was encouragement. Then I knew I could always reach out to [Name of Community Navigator] and then get back on track with the goal. So it was quite positive, whereas before I didn’t have even a goal set in my head. I was just going from day to day.”* (CN24)

*“I mean maybe, I don’t know, but maybe a lot of my goals in life, not just necessarily making friends and being more sociable, maybe other goals I have in life are very hard to achieve unless something miraculous happens.”* (CN04)

#### Accessible resources locally

The programme had a focus on connecting service users in to social activities and groups which could be sustained beyond the end of the programme, so practical considerations also shaped people’s ability to participate. Even in a large city where the study took place, in which opportunities are more plentiful than in other areas, a key influence was how manageable it felt to reach a particular location. People who were less confident with going out and using public transport, as well as people with a physical disability, particularly struggled if a place was not familiar or convenient to reach. It was therefore crucial that the Community Navigators responded to people’s individual needs, researching opportunities within an area that the person felt was realistically accessible, offering guidance on travel options, and in particular, accompanying people until they felt comfortable to travel somewhere on their own.

*“Yeah, if it was more local. Yes, it’s miles away. I don’t know the area. If I’d known the area a little bit better I might have gone, but I didn’t”* (CN12)

*“We went for a couple of walks [in a park] and expanded my boundaries by going further. One of the problems I have is, why the agoraphobia gets so bad is visual noise. So if you’re in a place where there’s traffic and people and lots and lots of different types of noises, I begin to almost get tunnel vision. It gets too much for me and I become terrified of being outdoors” (*CN35*)*

Cost was also a factor. People wanted to find opportunities that they could afford on an ongoing basis. The availability of free activities in the city was seen as helpful, as was the £100 budget that was available to each participant in the programme, to facilitate social contact. This enabled some to sign up to ongoing activities, which would have otherwise been unaffordable. However, for others, this budget was still not enough to enable them to pursue their interests.

*“I certainly wouldn’t have been looking forward to going to the [community centre], and…the sewing and the gardening – no way. And then I’ve got my gardening tools as well... For the money paid for that, I wouldn’t have been able to afford them myself.”* (CN31)

*“Because the courses were way out of budget. They were so expensive. To pay for a level three course or a level two course was hundreds and hundreds of pounds… that wasn’t an option.”* (CN24)

A third issue around accessibility concerned how welcoming groups and activity providers were. Differences were described between those with friendly facilitators, who put people at ease from the start, and those that had complex sign-up processes. How welcoming the initial experience was could act as a major deterrent or facilitator. Again, people described how important it was that the Community Navigators could accompany them on their first visits to a group or activity, giving them the confidence to enter a group setting.

*“She made us a cup of tea and gave us some biscuits, and we sat down, and she explained what they do…. And she said that we were quite welcome to go along.”* (CN31)

*“I remember when I went to enrol on one of the occasions, like I said, I had to go back several times and I went with different people, the person that was trying to enrol me, she was rejecting all my evidence for some reason…and I just had a massive panic attack.”* (CN15)

#### Timing of programme

When addressing loneliness in a group of people with severe depression and/or anxiety the emergence of ‘timing’ as a crucial influence was unsurprising. The programme came at the ‘right’ time for some people, when they were ready for things to change in their life, and were willing to give new things a go, even if this might be challenging for them. This readiness could be supported by the work of other mental health professionals, who might help people to prepare for taking part in the programme, thus allowing people to make the most use of it when it began.

*“Just I was at my lowest ebb when I got this phone call out of the blue. I thought ‘oh, well, this is something I can go with.’ I spoke to this woman and she sent someone round. So I thought ‘yes, I can do this. It will give me something to occupy my mind and maybe I’ll benefit from it.’*” (CN12)

*“I think for me, it was a pretty good time, because with all the counselling, I was, kind of, ready for all that stuff. I think for someone with a lot more anxiety and that kind of thing, would have trouble…Yes someone with more anxiety about simply going to somewhere new and stuff, probably would have a lot more trouble.”* (CN25)

In contrast, for others the timing was ‘wrong’, often because of having other priorities to deal with, such as serious physical health problems, or sitting tests at college, or because they were feeling too low to engage with the Community Navigator. Where service users felt too low, they often put off seeing their Community Navigator, or could not go ahead with the plans they had made for a session, ultimately limiting what they got out of the programme.

*“Because my mood is up and down and sometimes when [they] was trying to, I don’t know, take me out, I wasn’t feel to go out and even when [they were] here, I wasn’t feel to talk or do anything, you know what I mean?”* (CN21)

*“I felt like I could do more. But when it came around, I felt in a lower place. I didn’t feel like I could do as much in my head before when I said this Community Navigator thing where I could go places and stuff.”* (CN38)

Service users also spoke about how the six-month timeframe and limited number of sessions had restricted what they got out of the programme. It did not allow for the ‘downs’ that they experienced during the programme, or for the initial time it took for the Community Navigators to build their trust.

*“[They] didn’t give up on me. So I wish now that… if ever they do it again, that they do… but perhaps there are quite a few people like me that, in the beginning, they felt, ‘Oh, what’s this? What’s this? I don’t think so. What are you after? What are you after?’”* (CN31)

*“[They] tried to get hold of me, [they] keep calling me, but like I said, I wasn’t able to do anything, I wasn’t even returning her calls, I was just sleeping. And then, when I felt ready to do things, time’s up*.” (CN21)

#### Relationship with community navigator

Unsurprisingly, getting on with the Community Navigator and developing a working relationship with them was a strong influence on participation. From the first session, people were working out if the Community Navigator was someone they found likeable and comfortable with. Where this was the case, it acted as an incentive to participate in the programme, but it could also have the opposite effect: one person ended their participation because they found their Community Navigator over enthusiastic and pushed them too quickly.

*“The first meeting when I met my Community Navigator, it was quite daunting meeting someone new for the first time but [they] put me at ease. It was so relaxed, the situation. We just got on really well from the first meeting.”* (CN18)

*“I thought, well, okay, [they are] enthusiastic, [they are] new and all the rest of it but I found that slightly kind of ugh … It kind of made me think, ‘Oh God, this isn't going to work.’”* (CN26)

The factors that helped a relationship to develop between a service user and their Community Navigator are not unique, exemplifying many of the principles of good practice for supporting someone with mental health problems, such as listening to people to understand their needs and preferences, giving people choice and not pressurising them to do things they do not want to do. It was clear, however, that the Community Navigators had invested time and attention into relationship building, by doing things such as texting or calling between sessions to check in with how they were doing and finding commonality between themselves and the service user. This acted to build trust and helped people to feel cared about, forming the basis from which they felt willing to go with their Community Navigator to try out social opportunities.

*“I don't think [they have] ever done anything that's made me feel unhappy or uncomfortable or under pressure or anything. I mean I could easily recommend [them] to anybody else.” (*CN04*)*

*“That we both like crime books, that we both like certain things. [Their] knowledge of the internet was more or less on the same level as mine, maybe a bit more. But I didn't feel so inadequate. Like when I started going to [name of place where IT courses took place], I felt more at ease because I knew that I was with somebody in the same position as me.” (*CN03*)*

One distinguishing feature of the interaction between Community Navigators and the person they were supporting was that it was based on positivity and enthusiasm, the idea of just ‘giving things a go’. Community Navigators worked with people to identify what mattered to them and how they could connect this in with doing something social. Because the Community Navigator could go with them to try things out, it gave them the motivation to give things a go, even if they might otherwise duck out because they felt too nervous or down to do so. The presence of the Navigator was very helpful in access to unfamiliar situations and calming apprehensions. For some participants the verbal encouragement was essential, but for others it was more about just having a familiar person present as a social anchor.

*“Then we went there a second time, actually I think, oh [they] couldn’t make it at that time and I had to go by myself and I met everyone there and talked to them.”* (CN25)

*“Yes. Because I just couldn’t, you know… and there was no way I would walk in here on my own. I’d lost my confidence. There was no way. But [name of Community Navigator] said, ‘We can do it together, [name of participant].’ And I thought ‘wow, you know’ ”. (*CN31*)*

## Discussion

### Main findings

From our data it was clear how hard it was for people to work with feelings of loneliness: addressing loneliness involved taking risks and doing things, such as being around others, that did not always feel comfortable. We also found participants reacted differently to their Community Navigator; one person required praise and confidence to progress, while another found working with their Community Navigator created a sense of security and safety to explore their local area. We found people reached different stages in addressing feelings of loneliness, and even where loneliness, depression or anxiety were still present, people had achieved personally significant changes.

We also identified six key factors from the thematic analysis that help explain our narrative findings; factors which could either help or hinder participation and their degree of influence could vary over the course of the programme. Some of the factors relate to the individual participant, such as their desire to connect to others and their level of social confidence, particularly in social situations. Others function predominantly at the interpersonal level, e.g. the participant’s relationship with their Community Navigator, or to structural factors such as the availability of local resources that are both accessible and affordable. To our knowledge, this is the first study that has looked at the way in which individual and contextual factors affect how people with severe depression and/or anxiety participate in a programme aiming to reduce loneliness.

### Findings in the context of previous research

Our findings relate to and have implications for four areas of existing research regarding loneliness and wellbeing: asset-based approaches; social identity theory; recovery models; and therapeutic alliance. These are discussed below.

Addressing loneliness is challenging and complex [21]. Reducing feelings of loneliness was noted by the experts by experience on our working group to require high levels of emotional energy and courage and involved personal costs. The intersection of these efforts with the symptoms of depression and anxiety is a challenge to manage. Our study findings support conclusions from previous research that addressing loneliness may require both practical and psychological changes, such as changes to routine, increasing access to activities, as well as directly focusing on social connections and their appraisal [24]. Many theories of emotional and social loneliness prioritise the appraisal of relationships [41] and do not cover a domain that emerged as important in our findings: that of the accessibility of resources locally. The Community Navigator programme is located within Mann et al.’s typology as a “supported socialisation” intervention [9]. Theoretically, the programme links well to social identity theory [42] and asset-based approaches [43] which are key constructs for understanding how and why the Community Navigators approach may achieve benefits for participants. This distinguishes it from programmes underpinned more directly on cognitive or behaviour change models, which are centred on cognitive appraisal, social skills and psycho-education approaches [44]. Our study suggests that addressing people’s subjective appraisal of their social world may often not be enough to address loneliness: people need information and practical support to access local social resources too. This covers practical issues such as going into open spaces, accessing groups, transport, money and a companion to go out with. We may need to view loneliness within a broader systemic context to move beyond its emotional attributes to also consider wider contextual issues. This supports the value of asset-based approaches in mental health [45] and resonates with the person-centred approach taken by the Community Navigators, which provided each person with a bespoke package to support their journey to addressing feelings of loneliness. Approaches will also need to consider that progress may not be linear and some barriers, particularly structural issues including poverty, will be hard to address long-term through a programme of individual support.

Identifying people’s interests and what generates ‘meaning’ for an individual was also central. Social identity theory [44] helps explain how people’s sense of self, self-esteem, belonging and positive social identities are related to positive health outcomes. The Community Navigator strove to enhance and build upon existing identities as well as encourage new meaning through an identity formation process. We noticed that some of the people in the programme had a strong sense of identity such as businessperson, mother, artist. For some participants, depression and anxiety had a ‘stronger’ explanatory impact on how they felt about social relationships than these social identities but there were instances of people re-establishing positive social identities through the support of the Community Navigator. As has been demonstrated for adults with psychological distress [46], social identity approaches may also hold promise as a way of addressing loneliness among people using secondary mental health services.

‘Recovery’ is often related to five key concepts [47]: connectedness; hope; identity; meaning in life; and, empowerment. We found the desire to connect with others varied and was a fundamental barrier to reducing loneliness when absent in narratives. People spoke about low confidence in talking to others, poor self-esteem including feeling they did not have much to offer relationships, and poor trust in people. The Community Navigators were tasked with supporting people to develop new social relationships or reconnect with past contacts. If the service user was very reluctant to meet people socially, the focus shifted to using the time to explore opportunities to take up activities and go to different places. These steps are known to stimulate small social interactions otherwise termed ‘weak ties’ within social networks [48] and can act as bridging social capital [49]. Where desire to connect with other people was higher, the relationship with the Community Navigator developed more quickly and supported quicker progress thorough the programme to researching opportunities locally and understanding how best to support behaviour change.

The importance of ‘therapeutic relationships’ [50] guided the recruitment of people into Community Navigator roles. Priorities within recruitment included collaboration, empathy and respect in provider-client relationships as well as qualities found in peer support workers such as building trust [51] and appropriate use of personal disclosure [52]. The relationship with the Community Navigator emerged as a central influence on participation in the programme, with the personal qualities of the Community Navigator valued and acknowledged as well as the quality of relationship. We found that key Community Navigator characteristics valued by service users in the programme were flexibility of approach, authenticity in the relationship, sharing personal views and information, and using an encouraging, solution-focused approach. The approach was not judgements over ‘success’ and ‘failure’ but the role of Community Navigators was to support context specific progression. Progress included opening the door and conversing with the Community Navigator as well as for others more visible progress such as joining a new activity group.

Community Navigators acknowledged how hard it was in the context of health problems including mental health and sleep issues, as well as pervasive life difficulties such as relationship breakdown, financial concerns, immigration and housing insecurity to work with feelings of loneliness. The dedicated support could not always overcome other deeply embedded problems that some of those we worked with experienced. Although everyone interviewed signed up to take part in a social programme to address loneliness, not everyone was able to fully participate with their Community Navigator.

### Strengths and limitations

The co-produced approach to the design, delivery, analysis and write-up of this study was one of its strengths allowing expertise from experiential, clinical and academic experience to shape the study and its findings. The analysis process was rigorous, involving multiple readings of transcripts and time for discussion of codes and themes to come to a shared agreement about meaning. We have endeavoured to be transparent in the methods underpinning our analysis and in the evidence illustrating our results, to increase the credibility and trustworthiness of our findings [53]. The novel two-stage analysis approach was chosen to both keep a focus on the whole person and their individual story, consistent with the programme’s person-centred approach, whilst also searching for common themes across the data set.

Although we spoke to many of the people who took part in the feasibility study, our findings did not include those who we could not contact or did not consent to an interview. These individuals may have faced other challenges to participating in the programme. We also only worked in two NHS Trusts in inner and outer-London metropolitan environments, so would need further research to explore loneliness programmes in rural settings, where the range of options for social engagement may be more limited and transport is more of a challenge. This paper explores the views of programme participants: we acknowledge that other stakeholders, including the Community navigators, and involved family and mental health staff, might also have useful perspectives on factors influencing participation in the programme. A summary of stakeholders’ views on the acceptability of the programme has been reported elsewhere [28].

### Implications for research

While more research is still needed to fully understand the experience and drivers of loneliness for people with mental health problems, it is also a priority to develop and test of interventions which can alleviate loneliness, informed by current evidence, theory and first-hand knowledge.

This study has underlined the importance of researchers considering not only whether an intervention is effective, but also understanding what may affect the achievement of successful outcomes, especially in an area such as loneliness where human factors may affect intervention delivery in unanticipated ways. Future evaluations should include process evaluation components and areas that are rural or more asset poor, to investigate how such a programme would work where social resources are less widely or immediately available. Our study indicates several potential moderators and mechanisms of the effect of the Community Navigator programme, which we recommend could usefully be measured in a future trial to understand how the intervention may be reducing loneliness and improving health outcomes: these include: the therapeutic alliance, perceived self-efficacy; extent of positive social identities, and perceived neighbourhood social capital.

The many positive appraisals of the Community Navigator programme suggest a socially focused, asset-based approach to reducing loneliness may be suitable for people with severe anxiety or depression using secondary mental health services. We do not yet have definitive evidence regarding its effectiveness for reducing loneliness and improving health outcomes however: a larger research trial is recommended involving multiple sites. This could also allow for exploration of intervention effectiveness in different population sub-groups, and different geographical settings.

### Implications for practice

Addressing loneliness is both difficult and essential for wellbeing: people who are lonely tend to be more critical of others and themselves, be more self-conscious in social situations, and enter relationships with greater mistrust. However, our study shows that, despite internal and external barriers, people with severe depression and anxiety want help and can take steps to address loneliness. Some people will need more extensive work to address their own internal challenges to getting involved. Consideration should thus be given to who is offered a programme to address loneliness, to check they have sufficient desire to develop social connections at that point in time. Individuals may have other goals, such as gaining a new qualification, where other forms of support could better assist. Reviewing the six factors influencing participation may be one way to explore suitability for the programme; we urge flexibility in doing so, using the factors as a guide not a checklist.

It is also important to consider how people can be prepared to make the most of a time-limited loneliness intervention, for instance through conversations to identify interests and goals prior to taking part or supporting people to become more used to leaving their homes. Another element of timing is being able to take a pause in the programme when fluctuating mental health or other life factors make engagement difficult. There was limited opportunity for this within the constraints of a short-term research trial, but where possible, this should be considered for loneliness programmes offered as part of an ongoing clinical service.

## Conclusions

We found people with severe depression and/or anxiety, supported by secondary mental health services, may want to address feelings of loneliness but are aware of the emotional effort this may entail. How possible it is for people to participate in a guided support programme will vary, some people being very enthusiastic, others struggling to motivate themselves to achieve any engagement. Impacts will also be differently defined, and progress is unlikely to be linear. Interventions to address loneliness should be designed to take into account the factors that both enable and hinder participation, building in significant person-centred support structures to address these factors and recognising that their influence may fluctuate over time.

## Data Availability

We do not have consent to share full transcripts. The analysis data tables used during the current study are available from the study Principle Investigator at UCL – Dr. Lloyd-Evans on reasonable request.

## Abbreviations

NHS: National Health Service
NIHR: National Institute of Health Research
